# Guizhi Gancao Longgu Muli decoction for insomnia: A protocol for a systematic review

**DOI:** 10.1097/MD.0000000000019198

**Published:** 2020-02-28

**Authors:** Fangying Chen, Guoming Chen, Ziyin Chen, Zhaoping Zhang, Peng Zhang, Dongqiang Luo, Keyi Li, Yingyue Hou, Wanli Xing, Peiyu Shi, Xueya Yuan

**Affiliations:** a The First Affiliated Hospital of Guangzhou University of Chinese Medicine; b Guangzhou University of Chinese Medicine, Guangzhou, China.

**Keywords:** Guizhi Gancao Longgu Muli Decoction, insomnia, study protocol, systematic review

## Abstract

**Background:**

Insomnia is a prevalent and bothersome disorder of sleep initiation and maintenance. Although efficacious treatments for insomnia have been available for decades, they all have their own limitations. Guizhi Gancao Longgu Muli Decoction (GGLMD), a popular complementary and alternative therapy, has been widely applied to treat insomnia in some Asian countries for centuries. Yet no systematic reviews have comprehensively assessed the efficacy and safety of GGLMD as a treatment for insomnia.

**Methods:**

A comprehensive search up to November, 2019 will be conducted in the following electronic databases: the Cochrane Library, Embase, PubMed, Web of Science, the Chinese National Knowledge Infrastructure (CNKI), the Chinese Biomedical Literature Database (CBM), the Chinese Scientific Journal Database (VIP), and the Wanfang Database. The primary outcomes will be sleep quality including Pittsburgh Sleep Quality Index (PSQI) and polysomnography (PSG). Stata 15 will be used for data analysis as well.

**Results:**

This study will provide the current evidence of insomnia treated with GGLMD from the several points including PSQI and PSG.

**Conclusion:**

The consequence of this summary will furnish proof to evaluate if GGLMD is effective in the treatment of insomnia.

**Ethics and dissemination:**

Without personal information involved, ethical approval and informed consent form is no need. The review will be submitted to a peer-reviewed journal prospectively to spread our findings.

**PROSPERO registration number:**

PROSPERO CRD42018118336.

## 1 Introduction

### 1.1 Rationale of the condition

Insomnia is a prevalent complaint of sleep quantity or quality, manifesting as difficulty initiating or maintaining sleep, or difficulty falling asleep again after early awakening, which leads to daytime dysfunctions and a high risk of depression and anxiety.^[1,2]^ It is defined as chronic when these symptoms appear more than three nights a week for at least three months. The prevalence of insomnia is around 10% to 33%, and even higher in elders and women in the menopausal transition.^[3–5]^ Given the increasing prevalence and its bothersome symptoms, insomnia is being a huge burden of health and economy for patients themselves and society as well.^[6,7]^

Although the causes and pathophysiology of insomnia still remain complex and unclear, effective approaches to its treatment have been available for decades.^[4,8]^ Cognitive behavioral therapy (CBT-I) and pharmacotherapy (e.g., benzodiazepine receptor agonists) are the two major ones.^[9]^ The former is recommended as the first line therapy, while the more commonly used one is the latter.^[10]^ However, the treatment of insomnia stays challenging because both pharmacotherapy and non-pharmacologic options have their limitations (e.g., problems of accessibility, risks of cognitive and behavioral changes).^[11–13]^ Therefore, continued efforts are required to develop complementary and alternative therapies to help optimize the treatment. Recently, more systematic reviews about Chinese herbal medicine (CHM) in the treatment of insomnia have been performed, regarding all types of CHM or a specific type, which have suggested CHM as a well-tolerated and promising therapy for improving sleep disorders.^[14–17]^

### 1.2 Rationale of the intervention

Guizhi Gancao Longgu Muli Decoction (GGLMD) is a traditionally used herbal formula for insomnia, whose herbs consist of Cinnamomi Ramulus (cassia twig, guizhi), Glycyrrhizae Radix Et Rhizoma (licorice, gancao), Os Draconis (longgu), and Ostreae Concha (oyster shell, muli). Clinical studies have also reported the significant reduction of the Pittsburgh Sleep Quality Index (PSQI) score in GGLMD group, as well as indicated the great efficacy and well tolerance of GGLMD.^[18]^ Furthermore, the pharmacological researches have shown that the underlying mechanisms for its sedative-hypnotic effect may be mediated by regulating neurotransmission, with licorice modulating GABAA-BZD receptors and 5-HT2C receptors, Os Draconis (longgu), and Ostreae Concha (muli) enduringly eliciting antidepressant activity, etc.^[17,19,20]^ Despite the popular use of GGLMD, no systematic reviews have ever been planned or performed to evaluate its efficacy and safety.

## 2 Objectives

This study is performed to provide an assessment of the effectiveness and safety of GGLMD for insomnia, considering the evidence from related clinical randomized controlled trials (RCTs).

## 3 Method

The procedure of the protocol will be developed in compliance with the Preferred Reporting Items for Systematic Review and Meta-Analysis Protocols (PRISMA-P) 2015 statement. This protocol has been registered on PROSPERO (ID: CRD42018118336).

### 3.1 Criteria

#### 3.1.1 Types of studies

It is RCTs that we will include in order to provide more unbiased information. Editorials, observational cohort and case-control studies will be excluded. Cross-over trials will also be rejected owing to the possibility of a carry-over effect. Instead of written in English or Chinese, articles written in other languages will be excluded. We will not impose any restriction on publication status.

#### 3.1.2 Types of participants

We will enroll patients suffering from insomnia defined by the standard diagnosis criteria^[20]^ from European Sleep Research Society (ESRS) irrespective of age, sex or ethnicity. Patients combined with severe illnesses (e.g., cardiovascular and cerebrovascular diseases, respiratory diseases, infectious diseases or tumours) will be rejected.

#### 3.1.3 Types of interventions

Qualified interventions will be those involving GGLMD monotherapy in contrast to conventional western medicine treatment irrespective of dosing frequency and duration. Interventions including other Traditional Chinese medicine (TCM) treatment like acupuncture and moxibustion, cupping or massage will be rejected. GGLMD is constituted by Cinnamomi Ramulus (gui zhi), Glycyrrhizae Radix Et Rhizoma (gan cao), Keel (long gu), and Ostreae Concha (mu li). The plus and minus of GGLMD should contain the herbs above and adhere to the principles of formulating prescription.

#### 3.1.4 Types of outcome measures

Primary outcomes: sleep quality, including PSQI and polysomnography (PSG).

Secondary outcomes:

(1) successfully withdrawal rate,
(2) improvement of clinical symptoms,
(3) rate of adverse reactions.

### 3.2 Electronic searches

Without temporal limit, the following databases will be systematically searched to find eligible articles: the Cochrane Library, Embase, PubMed, Web of Science, the Chinese National Knowledge Infrastructure (CNKI), the Chinese Biomedical Literature Database (CBM), the Chinese Scientific Journal Database (VIP), and the Wanfang Database. The search strategy will be developed using the keywords listed below: “insomnia” and “Guizhi Gancao Longgu Muli decoction”. The proposed search strategy for PubMed has been provided in Table 1.

**Table 1:**
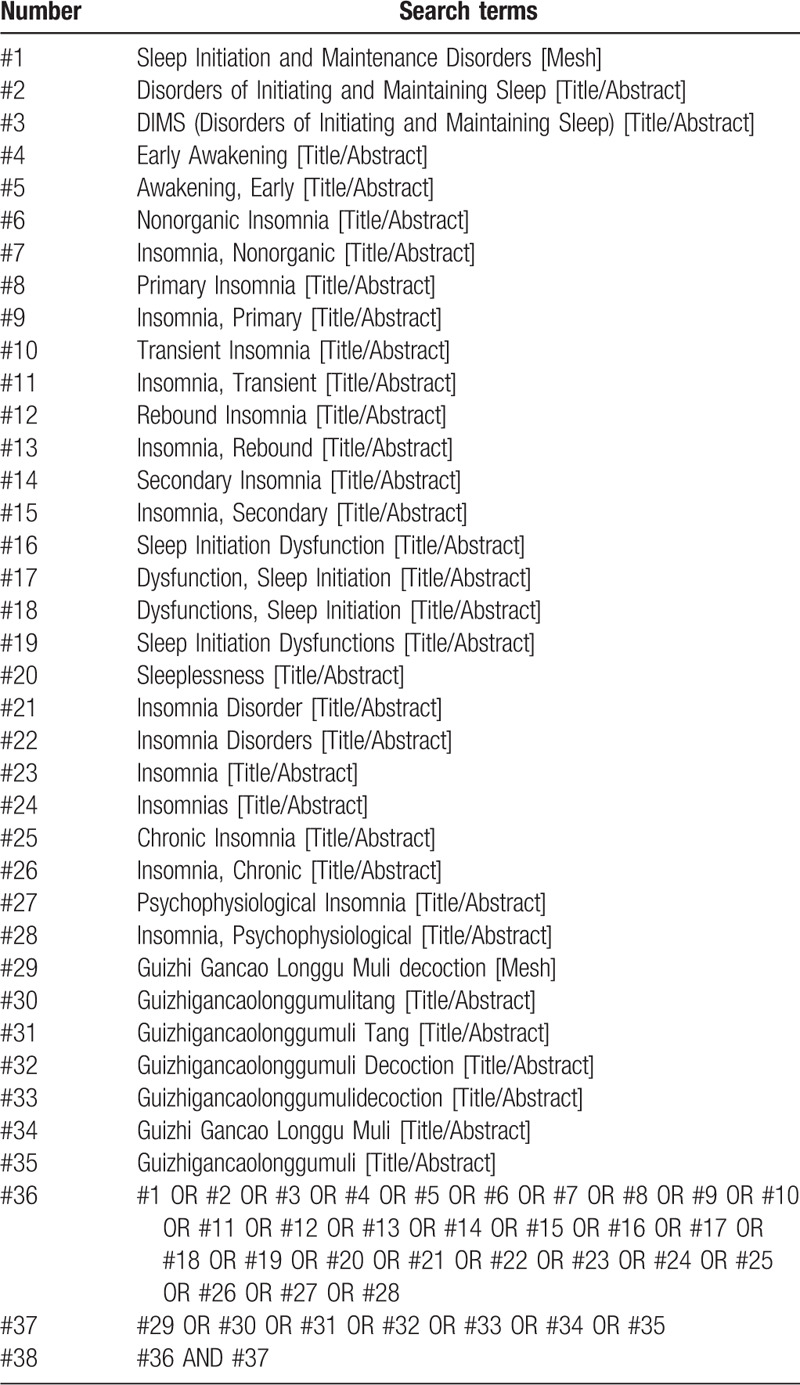
Search strategy for the PubMed database.

Moreover, the reference lists of relevant RCTs and systematic reviews will be hand-searched to retrieve more related studies.

### 3.3 Selection of eligible studies

After importing the search results to the EndNote X9, we will remove the duplicates. For preliminary study selection, all of the titles and abstracts will be screened and evaluated on the basis of the preestablished inclusion and exclusion criteria by two verifiers (PZ and DL) independently. After filtering evidently inappropriate studies, the full text of the rest will be reviewed for eligibility and may be rejected according to the criteria below:

(1) Not RCTs,
(2) RCTs but in inconformity with the inclusion criteria,
(3) Non-conforming intervention.

**Figure 1:**
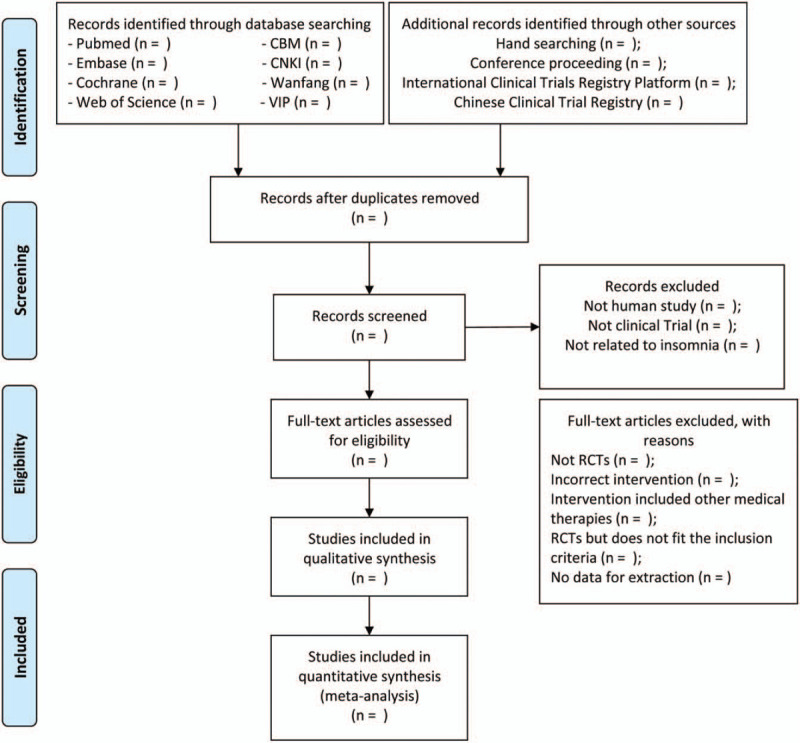
PRISMA flow diagram to describe the search process. PRISMA = Preferred Reporting Items for Systematic Reviews and Meta-Analyses.

In this phase, discussions will be of the most importance to solve the disagreements. Where consensus cannot be reached, the third investigator (KL) will participate in discussions and make an arbitration. Concrete reasons for exclusions will be listed and validated by the three investigators. Detailed screening process is shown in Figure 1.

### 3.4 Data extraction and management

Two review authors (DL and KL) working independently will implement the data extraction using an acquisition form developed by the first author. Data elements to be extracted include: type of studies, baseline patient characteristics, intervention measures, outcomes, and adverse events. Any discrepancy of study identification will be discussed and resolved by consensus. Otherwise, the third investigator (PZ) will make a final decision. Missing data will be supplemented through contacting the authors. If the data is unavailable, the study will be eliminated and the potential influence of missing data will be incorporated into the consideration on the results of meta-analysis.

### 3.5 Assessment of risk of bias

Risk of bias will be evaluated and defined as “low”, “high”, or “unclear” in accordance with the criterion of the Cochrane Handbook for Systematic Review of Interventions V.5.1.0 (updated March 2011)^[21]^ by two estimators independently. Generally, the following aspects will be taken into consideration for the quality assessment:

(1) the validity of sequence generation,
(2) blinding of participants, therapists and assessors,
(3) concealed allocation,
(4) deficient data,
(5) outcomes blinding,
(6) selective reporting,
(7) other sources of bias.

In the event of divergence, discussions will be held to come to an agreement.

### 3.6 Data analysis

Through the use of the Stata 15, data analysis will be effectuated. The mean difference (MD) for continuous data and risk ratios (RRs) or odds ratios (ORs) for dichotomous outcomes with 95% confidence interval (CI) will reflect the overall effect sizes.

### 3.7 Heterogeneity

Heterogeneity among the studies will be measured by calculating the *I*^2^ and *χ*^2^ statistics. *I*^2^ ranging from 50% to 100% will be interpreted as significant amount of heterogeneity. Fixed effect models will be used in the absence of obvious heterogeneity, or the random-effect will be applied.

### 3.8 Assessment of reporting bias

Through the construction of funnel plots, reporting bias will be detected by asymmetry, which will be evaluated using Begg and Egger tests.

### 3.9 Sensitivity analysis

Sensitivity analysis will be effectuated to verify the credibility of consequence and find whether there are any individual studies resulting in an obvious inconsistency. In order to figure out why, we will scrutinize the individual studies.

### 3.10 Subgroup analysis

For the sake of discovering latent sources of heterogeneity, we will conduct the subgroup analysis according to the course or dosage of herbal medicine treatment, sample size and the severity of the disease.

### 3.11 Quality of evidence

For purpose of rating the certainty of evidence (high, moderate, low, or very low), the Grading of Recommendations Assessment, Development, and Evaluation (GRADE) will be used. The quality of evidence will reduce to a lower rank owing to deviation, unexplained heterogeneity, limitations in study design, inexactness, and publication bias.

### 3.12 Patient and public involvement

No patients or public were involved.

## 4 Discussion

Insomnia, one of the most common clinical symptoms, can be seen in many kinds of diseases and tell on people's health. It affects brain thinking, memory and brings about anxiety and depression, leading to many problems in people's work, study and social activities. For the moment, drugs used for insomnia in clinic are principally Hypnotics and Sedatives, among which benzodiazepines (BZDs) are most frequently used.^[22]^ This class of medicine can bring about a series of side effects like rebound insomnia and withdrawal symptoms.^[23]^ Instead, non-benzodiazepines (non-BZDs) with less side effects have emerged to replace BZDs. But non-BZDs still get rise to some adverse reactions such as dizziness, headache, and amnesia. TCM has a long history with its distinct characteristics in the treatment of insomnia and has obtained more attention for its good efficacy and lower side effects. GGLMD, a prescription from Treatise on Febrile Diseases, has the effects of warming heart-yang and tranquilizing mind with heavy settling. It has a sedative impact on the central nervous system, effectively improving insomnia symptoms.

Until now, no systematic reviews reporting the effects of GGLMD on insomnia have been published. In consequence, this meta-analysis will be conducted with the view of providing recent evidence on the effectiveness and safety of GGLMD for insomnia. Particularly, some dosages and modifications will be identified through subgroups to detect more effective composition. We expect that the results of our study can provide a reference for researchers and physicians in clinical practice.

Nevertheless, our study is somewhat limited in the elements below. First, in the light of the criteria, articles written in other languages instead of English or Chinese will be excluded. Though it seems to result in insufficient information, it can be omitted because of limited number of these researches. Moreover, some studies are of poor quality because they make no mention about blinding and random methods, which will be evaluated by the risk of bias. Furthermore, some outcomes may be selectively reported, so we will be in strict accordance with the rule of systematic review to exclude the disqualifications.

## Author contributions

**Data curation:** Zhaoping Zhang, Peng Zhang, Dongqiang Luo, Peiyu Shi.

**Methodology:** Fangying Chen, Guoming Chen, Ziyin Chen, Keyi Li, Yingyue Hou, Wanli Xing.

**Supervision:** Fangying Chen, Guoming Chen, Ziyin Chen.

**Writing – original draft:** Fangying Chen, Guoming Chen, Ziyin Chen, Zhaoping Zhang, Peng Zhang, Dongqiang Luo, Keyi Li, Yingyue Hou, Wanli Xing, Peiyu Shi.

**Writing – review & editing:** Fangying Chen, Guoming Chen, Xueya Yuan.

Fangying Chen: 0000-0003-3134-6779.
